# Does Examining the Childhood Food Experiences Help to Better Understand Food Choices in Adulthood?

**DOI:** 10.3390/nu13030983

**Published:** 2021-03-18

**Authors:** Aleksandra Małachowska, Marzena Jeżewska-Zychowicz

**Affiliations:** Department of Food Market and Consumer Research, Institute of Human Nutrition Sciences, Warsaw University of Life Sciences (SGGW-WULS), Nowoursynowska 159C, 02-776 Warsaw, Poland; marzena_jezewska_zychowicz@sggw.edu.pl

**Keywords:** parental feeding practices, childhood recollections, eating behavior, validation study

## Abstract

Impact of parental feeding practices on children’s eating behaviors is well-documented in the literature. Nevertheless, little is known about how many of these behaviors might persist into adulthood. There is a lack of a tool measuring childhood feeding experiences recollected by adults, while the Comprehensive Feeding Practices Questionnaire (CFPQ) is used to measure parental feeding practices applied towards children. The aim of the study was to adapt the CFPQ to measure adults’ recollections of their childhood (5–10 years old) feeding experiences, to examine its discriminant validity and then to assess if these practices are related to adults’ food choices. In 2020, the modified version of CFPQ (mCFPQ) and questions on current food consumption were administered in a group of 500 adults twice over a two-week interval. The analysis included 443 participants whose questionnaires were correctly completed in both stages of the study. The Q-sorting procedure was used to test for discriminant validity of the questionnaire, i.e., confirmatory and exploratory factor analysis (EFA), Cronbach’s alpha, correlations coefficients, and the analysis of the differences between groups according to the intake of certain food products. Test–retest reliability was examined by calculating interclass correlation coefficients (ICC) for each obtained factor. As a result of EFA, five subscales were identified: “Restrictions”, “Healthy Eating Guidance”, “Pressure and Food Reward”, “Monitoring”, and “Child Control”. Items from these subscales created a new tool—Adults’ Memories of Feeding in Childhood (AMoFiC). Test for internal consistency, factor correlations, and discriminant validity proved satisfactory psychometric parameters of AMoFiC. “Pressure and Food Reward” and “Child Control” were associated with higher intake of sweets and salty snacks, whereas “Healthy Eating Guidance”, “Monitoring”, and “Restrictions” were associated with higher consumption of fresh fruits and vegetables. Despite the fact that the AMoFiC questionnaire requires further research, the findings of the study might be of practical use in counseling addressed to the parents.

## 1. Introduction

Food preferences shaped at a young age might persist into adolescence and then into adulthood, hence childhood is perceived as a critical moment for the development of future eating behaviors [1]. Negative childhood feeding experiences might disturb children’s sensitivity to internal hunger and satiety cues [2]. It may continue to have adverse impact and progress into maladaptive eating behaviors in adulthood, such as emotional eating [3,4]. 

In the family setting, a process of socialization based on the theory of social learning takes place [5]. Social learning is a mechanism in which environmental experiences, thus also those gathered in the family surroundings, are assimilated by an individual [6]. Parents, as major providers, models, and regulators in terms of food intake, influence children’s eating in the greatest manner and in a variety of ways [7]. Parental feeding practices (PFP) are defined as goal-directed, food-, or eating-related strategies used by the parents to influence their children’s eating manner, including what, when, and how much their child eats [8,9]. Studies have shown that PFP such as encouraging eating in a supportive manner, modeling favorable eating behaviors, eating meals together, and being responsive to children’s hunger and satiety signals might favor healthy eating behaviors in children, including higher intake of fruit and vegetables [10,11,12], lower intake of low-nutrient-dense foods [13], and greater diet quality [14]. 

Association between PFP and eating behaviors is more commonly investigated in early (birth to 6 years) and middle (6 to 12 years) childhood [8,15,16,17], in comparison to adolescence [18,19]. Available studies [3,4,20,21,22,23,24] indicate that childhood experiences might favor certain eating styles in adulthood (e.g., emotional eating, excessive food preoccupation, and disordered eating behaviors). However, there is a lack of research on the effect of PFP on food intake among young, middle-aged, and older adults [3,24]. Thus, the relationship between early feeding experiences, later eating behaviors, and food consumption still remains unclear and requires further examination [3]. Recognition of this association is limited by methodological aspects. Although longitudinal studies in this field could have been useful in explaining a causal relationship, they are problematic due to the prolonged period of observation and high risk of panel attrition [19,25]. In this case, retrospective studies are applied despite their limitations, such as lack of possibility to establish cause and effect or risk of recall bias [3,4,20,21,22,23,24]. Another problem results from the fact that there are currently no adequate tools to measure diverse childhood feeding experiences of adults that would allow to determine the link between parental feeding practices and future eating behaviors among people from different cultural groups [4,20,22].

The aim of the study was two-fold: (1) to adapt the original version of the Comprehensive Feeding Practices Questionnaire (CFPQ) to measure recollections of parental feeding practices in childhood and check some of its psychometric properties, and (2) to determine a relationship between those practices recollected by adults with their current food choices. We hypothesize that some of the PFP (e.g., pressure, emotional feeding, and control) may be associated with greater intake of sweets and salty snacks in adulthood, whereas other PFP (e.g., teaching about nutrition, encouragement, modeling, and involvement) may be related to lower intake of sweets and salty snacks and higher intake of fruit and vegetables in adulthood. The findings of this study will contribute to a better understanding of the relationship between childhood experiences related to selected PFP and food choices, both favorable and adverse, in adulthood. We believe that the adopted tool would be useful for further investigation of the role of childhood food experiences when explaining eating behaviors in adulthood.

## 2. Materials and Methods

### 2.1. Study Design and Sample Collection

Data were collected in February 2020 through a cross-sectional quantitative survey. The questionnaire was administered to participants twice over a two-week interval to estimate the test–retest reliability. Recruitment and data collection were conducted by a professional market research agency using the CAWI (Computer-Assisted Web Interview) technique. The study sample was recruited from an e-panel counting around 60,000 registered individuals. Only people aged 18–65 years old were included in the study. Quota controls including gender, age, place of residence, and region were set to obtain a representative sample of the Polish population. The study involved 500 participants. All participants gave voluntary consent to participate in both parts of the study in the form of a written informed consent. As 57 people did not respond to the invitation sent after two weeks to re-participate in the study, the final sample consisted of 443 participants.

### 2.2. Feeding Practices

The Comprehensive Feeding Practices Questionnaire (CFPQ) [26] was designed as a parent-report measure of feeding practices in children aged 2–8 years. It contains items distributed into 12 subscales (“Encourage Balance and Variety”, “Environment”, “Involvement”, “Modeling”, “Monitoring”, “Teaching About Nutrition”, “Emotion Regulation”, “Food as Reward”, “Pressure”, “Child Control”, “Restriction for Health”, and “Restriction for Weight Control”). In the study, a modified version of CFPQ (mCFPQ) was developed to enable retrospective reports of child feeding practices among adults, for example, we use” My parents encouraged me to eat less so I won’t get fat” instead of “I encourage my child to eat less so he/she won’t get fat” from the original version of the CFPQ. Respondents were asked to report how frequently different situations took place in their childhood, using a 6-point scale: 1: “never”, 2: “rarely”, 3: “sometimes”, 4: “mostly”, 5: “always”, 6: “I don’t remember”. Moreover, they related to the sentences describing family habits from the period of their childhood using a 6-point scale: 1: “disagree”, 2: “slightly disagree”, 3: “neither agree nor disagree”, 4: “slightly agree”, 5: “agree”, 6: “I don’t remember”. The answer “I don’t remember” was added to minimize the risk of recall errors. The mCFPQ was transculturally adapted by translating it into Polish and conducting the pre-test in the group of 89 students at the beginning of the Nutritional Sociology course. Then, for the purpose of this paper, it was translated back into English. The participants were asked to reflect on their recollections from the age between 5 and 10 years old [4]. Middle childhood was selected as it represents a stage when children become more autonomous with their eating habits. Moreover, it is more probable for adults to recall memories from this period of time rather than from the earlier childhood [20].

### 2.3. Food Intake

Intake of 5 food groups, i.e., vegetables and fruits (separately fresh and processed), fruit/vegetable or mixed juices, sweets, and salty snacks was assessed using questions derived from the Dietary Habits and Nutrition Beliefs Questionnaire (KomPAN^®^) [27]. Respondents reported on their food consumption using a 6-point frequency scale ranging from ‘never’ (1) to ‘few times a day’ (6). Those categories were converted into values reflecting daily frequency of consumption for each food group (the range: 0–2 times/day) [28]. Participants were also asked how many portions of products from each food group they eat per day given that 1 portion of vegetables and fruit (fresh and processed) equals 100 g, 1 portion of juice equals 100 milliliters, and 1 portion of sweets and salty snacks equals 50 g. Examples of the portion sizes were added for each question. Food intake was calculated for each food group by multiplying daily frequency of consumption and amounts of portions consumed.

### 2.4. Statistical Analysis

Frequency analysis was performed to present sociodemographic characteristics of the study sample. 

The Q-sorting procedure was used for testing the discriminant validity concerning a substantive and a structural component of construct validity [29]. This procedure helps to separate items in a multi-dimensional construct and to eliminate items that do not discriminate well between categories of items [30]. Both exploratory and confirmatory methods were applied. Since the original factor structure of CFPQ [26] was not replicated, exploratory factor analysis (EFA) was conducted. Items from mCFPQ were treated as ordinal. The following criteria were selected to determine the final number of factors: components with an eigenvalue of 1, a scree plot test, and the interpretability of the factors. Information sources with factor loadings of at least 0.5 were taken into account. The factorability of the data was confirmed with the Kaiser–Meyer–Olkin (KMO) measure of sampling adequacy and Bartlett’s test of sphericity. The internal consistency of items within each identified factor was tested using Cronbach’s alpha, with values higher than 0.70 considered acceptable. The Kolmogorov–Smirnov test was used to test the normality of each factor. As distributions were found to be non-normal, Spearman’s correlations were applied to check for overlap between factors, given that correlation values r ≥ 0.85 are indicative of a strong overlap [31]. 

To evaluate an instruments’ discrimination capability, the relationships between the identified subscales (factors) and selected food products’ intake were applied [29]. Mann–Whitney’s test was used to compare mean values of each identified factor within the intake of five food groups, including (1) sweets and salty snacks, (2) fresh fruits and vegetables, (3) processed fruits and vegetables, (4) vegetable, fruit, and mixed juices, and (5) total fruits and vegetables. Within each group of food products, two categories of intake were identified using median value, i.e., “low” intake—below median, and “high” intake—above median. 

Assessment of test–retest reliability was conducted by calculating interclass correlation coefficients (ICC) for each identified factor (subscale). ICC values for subscales greater than 0.40 were considered reliable.

Statistical analysis was conducted using IBM SPSS Statistics for Windows, version 26.0 (IBM Corp, Armonk, NY, USA).

## 3. Results

### 3.1. Characteristics of the Study Sample

Characteristics of the study sample are presented in Table 1. Mean age of participants was 42.4 years (±13.0 standard deviation (SD)).

### 3.2. Structure of Modified Comprehensive Feeding Practices Questionnaire (mCFPQ)

Exploratory factors analysis revealed a factor structure consisting of 5 subscales, which were named as: “Restrictions” (13 items), “Healthy Eating Guidance” (9 items), “Pressure and Food Reward” (6 items), “Monitoring” (5 items), and “Child Control” (6 items). The factor-loadings, Cronbach’s alphas, and % of variance explained are presented in Table 2. The KMO value was found to be 0.965, and Bartlett’s test was significant at *p* < 0.0001 [31]. Loadings equal to 0.50 or higher were used to identify the components of the factors. Out of 49 original items, 13 were excluded (3 items—“Restriction for Health”, 2 items—“Emotion Regulation”, 2 items—“Environment”, 2 items—“Modeling”, 1 item—“Teaching about Nutrition”). It resulted in a 39-item questionnaire (Table 2), which suggested name is Adults’ Memories of Feeding in Childhood (AMoFiC). 

Table 3 presents Spearman’s correlations between identified factors. No significant correlations were found between variables.

### 3.3. The Relationships between the Subscales of the Adults’ Memories of Feeding in Childhood (AMoFiC) and Food Consumption 

Fresh fruits and vegetables were consumed at least once a day, 46% and 33.6%, respectively (Table 4). The majority of respondents consumed processed fruits and vegetables less than once a day (91.6% and 83.3%, respectively). Less than 1/5 of the study sample consumed vegetable, fruit, and mixed juices at least once a day. Over 1/5 of participants ate sweets at least once a day, while 7.2% ate salty snacks with such frequency. Fresh fruits were the most consumed food (2.0 ± 2.3 portions per day), whereas vegetable, fruit, and mixed juices were the least consumed foods (0.1 ± 1.6 portions per day).

Results of discriminant capability of the AMoFiC are presented in Table 5. Higher intake of sweets and salty snacks in adulthood was associated with higher scores for feeding practices in childhood included in “Pressure and Food Reward” and “Child Control” factors. Higher intake of fresh fruits and vegetables in adulthood was associated with higher scores for parental feeding practices included in “Restrictions”, “Healthy Eating Guidance”, and “Monitoring” factors. Higher intake of processed fruits and vegetables in adulthood was associated with higher scores for parental feeding practices included in “Healthy Eating Guidance” and “Monitoring” factors. At the same time, consumption of vegetable, fruit, and mixed juices was positively associated with scores for all identified factors. However, total consumption of fruits and vegetables was associated positively with parental feeding practices included in “Healthy Eating Guidance” and “Monitoring”.

Assessment of test–retest reliability revealed the following ICC values for each newly identified factor: “Restrictions”—0.674, “Healthy Eating Guidance”—0.668, “Pressure and Food Reward”—0.651, “Monitoring”—0.634, and “Child Control”—0.559.

## 4. Discussion

So far, the attempts to adopt the Comprehensive Feeding Practices Questionnaire (CFPQ) to measure adults’ childhood recollections were made, however, the study groups consisted solely of students and only selected subscales and questions from the original CFPQ were chosen [4,20,22]. We adopted a full version of the questionnaire. As a result of the exploratory factor analysis, we obtained a 39-item Adults’ Memories of Feeding in Childhood (AMoFiC) questionnaire with satisfactory psychometric parameters, including acceptable Cronbach’s alpha values, test–retest reliability, discriminant validity, and lack of statistically significant correlations between newly identified factors. However, psychometric parameters of this measure should still be confirmed in future research among different study groups. Further testing of its discriminant and convergent validity is also required [32].

We confirmed the hypothesis that specific factors associated with feeding practices in childhood might determine food consumption in adulthood. Results provide evidence that greater intensity of practices characteristic for “Pressure and Food Reward” and “Child Control” might favor higher intake of sweets and salty snacks. Forced consumption in response to fussy eating may not only deepen food aversion, but also persist into adulthood, leading to lower intake of target foods and higher intake of unfavorable foods [22,24]. Rewarding with food to influence children’s behavior or as a mood enhancer might result in emotional eating in adulthood [4,20]. Emotional eating is characterized by eating mostly highly palatable foods, such as processed, high-energy, high-fat, and high-sugar food products, which may result in weight gain [33]. 

The study results suggest that parental control might be related to unhealthy eating behaviors in adulthood. Pressure to eat and restrictive feeding (overt control) can be easily detected by the children, whereas other practices such as buying healthy food and avoiding places selling unfavorable foods, being examples of covert control, are less likely to be recognized. Moderate control, including covert control, is believed to support healthier eating behaviors in children [1]. The AMoFiC “Child Control” factor did not take into account those two types of control separately. Moreover, after exploratory factor analysis, items describing “Environment” and “Modeling” factors, which could have been interpreted as examples of covert control, were excluded. Further research to determine if both covert and overt control might predispose to more frequent consumption of unfavorable foods is needed. 

As expected, “Healthy Eating Guidance” including setting a healthy environment, modeling food behaviors, and allowing child to participate in the process of choosing, buying, and preparing meals, was associated with higher consumption of fresh fruits and vegetables [32]. Similarly, “Monitoring” turned out to be associated with higher consumption of fresh fruits and vegetables. Practices associated with monitoring are based on observing children’s behavior without turning into intrusive of restrictive methods [1]. Whereas restrictive feeding might promote unhealthy eating behaviors. Foods which are limited might be viewed as a “forbidden fruit”, leading to excessive food preoccupation. Desire to eat those products in larger quantity when they are available might persist into adulthood, increasing chances of disordered eating, including emotional eating and binge eating [4]. Nevertheless, our study did not confirm the relationship between restrictions applied in childhood and high consumption of sweets and salty snacks in adulthood. One possible explanation might be that the intake of those unfavorable foods was fairly low among the study group. Moreover, recollections on eating sweets in childhood might have been associated with being rewarded for good behavior [23], which was confirmed by our results. “Restrictions” factor was found to be linked with higher fresh fruits and vegetables consumption [34]. However, promoting restrictive practices to increase children’s intake of those favorable food products cannot be accepted as parents may experience resistance while limiting healthy foods for their child. Nonetheless, the role of parental restrictive feeding on the intake of those foods in adulthood should be further studied. 

The current study provided evidence that intake of certain food groups in adulthood might be determined by parental feeding practices in childhood. More research with the use of AMoFiC and other tools is required to confirm this relationship with regard to the intake of both healthy and unhealthy food products and the usefulness of the tool developed in the current study. The previous research did not analyze the associations between parental feeding practices in childhood with food intake in adulthood, yet it examined how childhood experiences are correlated with eating styles in adulthood [4,20,22]. Thus, further studies concerning food consumption but also eating styles in adulthood are needed.

### Strengths and Limitations

To our knowledge, this study is the first to use a full version of the Comprehensive Feeding Practices Questionnaire (CFPQ) in adults to measure their childhood recollections. Moreover, our study attempted to fill the knowledge gap on the impact of childhood experiences associated with feeding practices on future food choices. The strength of this study is also the use of a representative study sample in terms of age, gender, education level, and place of residence, thus the outcomes can be generalized to the population.

Despite the mentioned advantages, the study has certain limitations. The findings of the study were based on retrospective self-reports, which could have been biased, for example by imprecise recollections or social-desirability bias [3]. Secondly, the data were collected among Polish adults, hence the results cannot be generalized to other populations due to the differences associated with ethnicity or socioeconomic status [20]. Moreover, as mentioned in the Introduction Section, the cross-sectional design of this study does not allow to find a causal relationship. Additionally, the measure of childhood recollections in adults used in our study (mCFPQ) has been rarely used in the previous studies, thus there is a need for future research to assess its psychometric properties and confirm its validity. We assessed test–retest reliability of the questionnaire and although reliability is an important aspect of measurement, it is not sufficient to confirm the validity of the test [35]. Moreover, there is no general consensus on what constitutes a good ICC [36,37]. Thus, further studies are required to provide insight into the test–retest reliability and validity of the questionnaire, especially including convergent validity.

Despite these limitations, the findings of this study may contribute to a better understanding of the relationship between childhood experiences related to selected parental feeding practices and food choices, both favorable and adverse, in adulthood.

## 5. Conclusions

The current study did not manage to confirm the original structure of the Comprehensive Feeding Practices Questionnaire (CFPQ) with the use of its modified version (mCFPQ); however, a newly developed shortened tool (AMoFiC) demonstrated good psychometric properties, proving that it might be used to measure retrospective reports of children’s feeding practices in adults, however further research to confirm its properties is still needed. The study results showed that childhood experiences might favor certain dietary patterns in adulthood, both adverse (snacking on sweets and salty snacks) and favorable (consumption of fruits and vegetables). Future research should focus on the impact of parental feeding practices on other dietary habits and diet quality and assess their relationship with eating styles. 

## Figures and Tables

**Table 1:** Socio-demographic characteristics of the study sample.

	*n* *	%
Gender		
Women	224	50.6
Men	219	49.4
Age (in years)		
18–24	40	9
25–39	149	33.6
40–54	142	32.1
55–65	112	25.3
Education		
Primary	68	15.3
Lower secondary	107	24.2
Upper secondary	156	35.2
Higher (e.g., BSc, MSc)	112	25.3
Place of residence		
Village	163	36.8
Town below 20,000 inhabitants	54	12.2
Town between 20,000 and 100,000 inhabitants	82	18.5
City over 100,000 inhabitants	144	32.5

* n—number of participants.

**Table 2:** Factors and items included in the modified Comprehensive Feeding Practices Questionnaire (mCFPQ).

Factors (Subscales) and Items	Factor Loadings **	Original Factor (CFPQ)
**Factor 1—Restrictions**		
18.* My parents took care of me not eating too many high-fat foods.	0.595	Restriction for Weight Control
27. My parents encouraged me to eat less so I won’t get fat.	0.778	Restriction for Weight Control
29. My parents gave me small helpings of food to control my body weight.	0.769	Restriction for Weight Control
31. My parents discussed with me the nutritional value of foods.	0.664	Teaching about Nutrition
33. If I ate more at one meal, my parents tried to decrease my food helpings at the next meal.	0.749	Restriction for Weight Control
34. My parents restricted the foods that would possibly make me gain weight.	0.688	Restriction for Weight Control
35. My parents believed that there are certain foods that I should not consume to prevent weight gain.	0.683	Restriction for Weight Control
36. My parents limited sweets/desserts for me in response to bad behavior.	0.588	Food reward
40. My parents wanted to be sure that I do not eat too much of my favorite foods.	0.557	Restriction for Health
41. My parents did not allow me to eat between meals because they didn’t want me to gain weight.	0.682	Restriction for Weight Control
42. My parents told me what I can and cannot eat without any explanation. **R**	0.610	Teaching about Nutrition
45. My parents have often put me on a diet to control my weight.	0.695	Restriction for Weight Control
48. My parents showed me how much they enjoy ‘healthy eating’.	0.553	Modeling
Cronbach’s Alpha	0.947	
% variance explained	41.5	
**Factor 2—Healthy Eating Guidance**		
14. Most of the foods that my parents kept in the house were ‘healthy’.	0.610	Environment
15. My parents involved me in planning family meals.	0.571	Involvement
20. My parents allowed me to help prepare family meals.	0.740	Involvement
22. As a child, I had access to many ‘healthy foods’ at each meal.	0.687	Environment
24. My parents encouraged me to try new foods.	0.693	Encourage Balance and Variety
26. My parents told me that ‘healthy food’ tastes good.	0.552	Encourage Balance and Variety
32. My parents encouraged me to participate in grocery shopping.	0.553	Involvement
38. My parents encouraged me to eat a variety of foods.	0.702	Encourage Balance and Variety
44. My parents modelled healthy eating for me by eating healthy foods themselves.	0.562	Modeling
Cronbach’s Alpha	0.902	
% variance explained	6.0	
**Factor 3—Pressure and Food Reward**		
17. My parents always insisted on finishing everything I had on the plate.	0.663	Pressure
19. My parents offered me my favorite foods in exchange for good behavior.	0.587	Food reward
23. My parents offered me sweets as a reward for good behavior.	0.610	Food reward
30. My parents insisted that I eat even though I told them that I’m not hungry.	0.664	Pressure
39. If I ate a small helping, my parents tried to get me to eat more.	0.683	Pressure
49. After finishing a meal, my parents tried to get me to eat more, even a bite of food.	0.617	Pressure
Cronbach’s Alpha	0.860	
% variance explained	4.9	
**Factor 4—Monitoring**		
1. Did your parents pay attention to the sweets that you were eating as a child?	0.742	Monitoring
2. Did your parents pay attention to the salty snacks that you were eating as a child?	0.729	Monitoring
3. Did your parents pay attention to the high-fat foods that you were eating as a child?	0.582	Monitoring
4. Did your parents pay attention to the sugary drinks that you were drinking as a child?	0.752	Monitoring
13. Did your parents encourage you to eat healthy foods before unhealthy ones?	0.635	Encourage Balance and Variety
Cronbach’s Alpha	0.862	
% variance explained	4.9	
**Factor 5—Child Control**		
5. Did your parents let you consume everything you wanted?	0.720	Child Control
6. Did your parents let you choose the foods you wanted from what was being served for dinner?	0.602	Child Control
7. Did your parents give you something to eat or drink as a first thing when you got fussy?	0.566	Emotion Regulation
10. Did your parents make something else when you did not like what was being served?	0.553	Child Control
11. Did you have access to snacks throughout the day?	0.694	Child Control
12. Did your parents allow you to leave the table when you were full even when the rest of the family was not done eating?	0.592	Child Control
Cronbach’s Alpha	0.787	
% variance explained	2.7	
**% total variance explained**	59.4	
Excluded items:		
8. Did your parents give you something to eat or drink when you were bored even though they knew you were not hungry?	-	Emotion Regulation
9. Did your parents give you something to eat or drink when you were upset even though they knew you were not hungry?	-	Emotion Regulation
16. There were a lot of salty snacks in my parents’ house. **R**	-	Environment
21. If my parents did not control my eating, I would have eaten more of my favorite foods.	-	Restriction for Health
25. My parents discussed with me why ‘eating healthy’ is important.	-	Teaching about Nutrition
28. If my parents did not control my eating, I would have eaten more ‘unhealthy foods’.	-	Restriction for Health
37. My parents kept a lot of sweets in the house. **R**	-	Environment
43. My parents wanted to be sure that I did not eat too many sweets.	-	Restriction for Health
47. My parents were enthusiastic about ‘healthy eating’.	-	Modeling
46. My parents ate ‘healthy foods’ in front of me even if they were not their favorite ones.	-	Modeling

* Number of statement from original CFPQ; ** correlation coefficient; items 1–13 utilize a 5-point scale: 1—“never”; 2—“rarely”; 3—“sometimes”; 4—“mostly”; 5—“always”; items 14–49 utilize a 5-point scale: 1—“disagree”; 2—“slightly disagree”; 3—“neither agree nor disagree”; 4—“slightly agree”; 5—agree”; items marked with **R** were reverse coded.

**Table 3:** Spearman’s correlations (rho) between identified factors.

Identified Factors	Identified Factors				
	Restrictions	Healthy Eating Guidelines	Pressure and Food Reward	Monitoring	Child Control
Restrictions	1.000	−0.033 (*p* = 0.490)	0.040 (*p* = 0.400)	0.048 (*p* = 0.317)	0.007 (*p* = 0.878)
Healthy Eating Guidance	-	1.000	0.022 (*p* = 0.642)	0.013 (*p* = 0.786)	−0.059 (*p* = 0.213)
Pressure and Food Reward	-	-	1.000	0.011 (*p* = 0.821)	0.007 (*p* = 0.890)
Monitoring	-	-	-	1.000	0.032 (*p* = 0.505)
Child Control	-	-	-	-	1.000

*p*—level of significance.

**Table 4:** Food consumption in the study sample.

	Frequency Consumption (%)		Number of Portions a Day (%)		Number of Portions a Day * (mean ± SD)
	Less Than Once a Day	At Least Once a Day	Less Than 1 Portion	At Least 1 Portion	
Fresh fruits	54.0	46.0	8.1	91.9	2.0 ± 2.3
Processed fruits	91.6	8.4	30.2	69.8	0.5 ± 1.1
Fresh vegetables	66.4	33.6	13.1	86.9	1.7 ± 2.2
Processed vegetables	83.3	16.7	8.8	91.2	1.0 ± 1.4
Vegetable, fruit, and mixed juices	85.6	14.4	25.1	74.9	0.1 ± 1.6
Sweets	78.8	21.2	20.5	79.5	1.0 ± 0.2
Salty snacks	92.8	7.2	10.4	89.6	0.7 ± 1.3

SD—standard deviation; * number of portions a day including daily frequency consumption.

**Table 5:** Discriminant capability for the Adults’ Memories of Feeding in Childhood using selected food products intake.

	Factors (Subscales)				
	Restrictions	Healthy Eating Guidance	Pressure and Food Reward	Monitoring	Child Control
	(mean ± SD)	(mean ± SD)	(mean ± SD)	(mean ± SD)	(mean ± SD)
Sweets and salty snacks					
Low intake ^a^ (M ≤ 0.8 portion a day)	2.3 ± 1.0	3.0 ± 1.0	2.6 ± 1.0 **	2.6 ± 1.1	2.5 ± 0.9 ***
High intake ^a^ (M > 0.8 portion a day)	2.4 ± 1.1	3.1 ± 0.9	2.8 ± 0.9 **	2.7 ± 1.0	2.8 ± 0.8 ***
Fresh fruits and vegetables					
Low intake (M ≤ 2.0 portion a day)	2.3 ± 0.9 *	2.9 ± 0.8 **	2.6 ± 0.9	2.6 ±1.0 *	2.7 ± 0.8
High intake (M > 2.0 portion a day)	2.5 ± 1.1 *	3.2 ± 1.0 **	2.7 ±1.1	2.8 ± 1.1 *	2.8 ± 0.9
Processed fruits and vegetables					
Low intake (M ≤ 1.0 portion a day)	2.3 ± 1.0	2.9 ± 1.0 *	2.6 ± 1.0	2.5 ± 1.0 ***	2.6 ± 0.8
High intake (M > 1.0 portion a day)	2.4 ± 1.0	3.1 ± 0.9 *	2.8 ± 0.9	2.9 ± 1.0 ***	2.8 ± 0.9
Vegetable, fruit, and mixed juices					
Low intake (M ≤ 0.3 portion a day)	2.2 ± 0.9 ***	2.9 ± 0.9 ***	2.5 ± 0.9 ***	2.5 ±1.0 ***	2.5 ± 0.8 ***
High intake (M > 0.3 portion a day)	2.6 ± 1.0 ***	3.3 ± 0.9 ***	2.9 ±1.0 ***	2.9 ± 1.0 ***	2.9 ± 0.9 ***
Total fruits and vegetables					
Low intake (M ≤ 4.0 portion a day)	2.3 ± 0.9	2.9 ± 0.8 **	2.6 ± 0.9	2.6 ±1.0 *	2.6 ± 0.8
High intake (M > 4.0 portion a day)	2.5 ± 1.1	3.2 ± 1.0 **	2.8 ±1.0	2.8 ± 1.1 *	2.8 ± 0.9

^a^ “low” intake—below median, and “high” intake—above median; * *p* < 0.05; ** *p* < 0.01; *** *p* < 0.001 (Mann–Whitney’s test); M—median; mean ± SD (standard deviation) based on a 5-point scales: 1—“never”/”disagree”; 2—“rarely”/”slightly disagree”; 3—“sometimes”/“neither agree nor disagree”; 4—“mostly”/”slightly agree”; 5—“always”/”agree”.

## Data Availability

The data presented in this study are available on request from the corresponding author.
